# Real-time locating and speed measurement of fibre fuse using optical frequency-domain reflectometry

**DOI:** 10.1038/srep25585

**Published:** 2016-05-05

**Authors:** Shoulin Jiang, Lin Ma, Xinyu Fan, Bin Wang, Zuyuan He

**Affiliations:** 1 State Key Laboratory of Advanced Optical Communication Systems and Networks, Shanghai Jiao Tong University, 800 Dongchuan Road, Minhang, Shanghai 200240, China

## Abstract

We propose and experimentally demonstrate real-time locating and speed measurement of fibre fuse by analysing the Doppler shift of reflected light using optical frequency-domain reflectometry (OFDR). Our method can detect the start of a fibre fuse within 200 ms which is equivalent to a propagation distance of about 10 cm in standard single-mode fibre. We successfully measured instantaneous speed of propagating fibre fuses and observed their subtle fluctuation owing to the laser power instability. The resolution achieved for speed measurement in our demonstration is 1 × 10^−3^ m/s. We studied the fibre fuse propagation speed dependence on the launched power in different fibres. Our method is promising for both real time fibre fuse monitoring and future studies on its propagation and termination.

Fibre fuse phenomenon is an optical discharge that may occur when power density of lightwave in an optical fibre exceeds a certain level^1^,^2^. Fibre fuse brings irreversible damage to the fibre core by leaving a series of voids^3^. The threshold power of fibre fuse is an essential parameter, which refers to the minimal laser power sustaining the fibre fuse propagation in this paper^4^. Threshold power of fibre fuse for a standard single-mode fibre (SSMF) is 1.3–1.5 W, which is sufficiently high for conventional telecommunication systems^5^,^6^. Some fibres with high Ge-dopant concentrations or small mode field diameters (MFD) have threshold powers lower than 1 W^4^. Transmission capacity of a single piece of optical fibre is increasing rapidly by employing technologies such as high power Raman amplification^7^, dense wavelength division multiplexing (DWDM)^8^ and space division multiplexing (SDM)^9^. Recently, 2.15 Pb/s transmission using a multi-core fibre and wideband optical comb has been reported^10^. However, the adoption of advanced amplification and multiplexing technologies inevitably results in a dramatic increase of optical power in a fibre. On the other hand, fibre laser technologies have also achieved a rapid progress in the past decade^11^,^12^. Fibre lasers with kW-order output power have become commercially available for industrial applications. Fibre fuse effect in a large-mode-area Yb-doped double-clad fibre with a launched power of about 400 W has been observed^13^. How to instantaneously detect and terminate the fibre fuse is becoming a realistic problem in both fibre communication and high power laser delivery applications.

The quick termination of a fibre fuse can largely reduce repairing difficulties of the damaged fibre. Moreover, an optical fibre damaged by the fibre fuse cannot be easily detected or identified from its appearance. It is critical to instantaneously detect/terminate the fibre fuse and accurately locate its position. Several termination techniques have been proposed by using cladding etched fibres, optical micro wires and hole-assisted fibres as fuse termination devices^14^,^15^,^16^. These techniques are used for termination of the fibre fuse without detecting its start and the fibre fuse does not stop until it reaches the termination device. Abedin *et al*. proposed a fibre fuse detection method by monitoring radio frequency (RF) spectrum of the backreflected light^17^. This method has difficulties in locating the fibre fuse and the RF signal is susceptible to the changes of transmission data rate and modulation format in the fibre. They have also proposed a fibre fuse monitoring method by detecting the weak reflection light from the fibre fuse by using an optical time-domain reflectometer (OTDR)^18^. This method allows detection of a fibre fuse at a remote location. However, it requires tens of seconds to make an average and the spatial resolution of OTDR is not sufficient for locating the damaged fibre.

On the other hand, the propagation speed is one of the important parameters of fibre fuse, which has been measured by using high-speed cameras and fibre Bragg grating (FBG) sensors^19^,^20^. However, there is still a lack of an efficient method to continuously measure the instantaneous speed of a propagating fibre fuse.

In this paper, we propose a technique of real-time monitoring of fibre fuse using optical frequency-domain reflectometry (OFDR). The proposed method mimics the operation of a Doppler radar. By bouncing a lightwave instead of a microwave signal off a moving target and analysing the frequency changes in the returned signal, instantaneous and highly accurate measuring on speed of the target can be realized. In the case of a propagating fibre fuse, the hot plasma moves towards the light source and the boundary of it causes a minute reflection of light which introduces a change in the beat frequency of OFDR owing to the Doppler shift. By analysing the acquired frequency information from OFDR we can accurately measure the propagation speed and the location of the plasma in real time. In experiment, we realized fibre fuse detection within 200 ms which is equivalent to a propagation distance about 10 cm in SSMFs. After the fibre fuse was terminated, the location of the last void in the damaged fibre was located with a resolution as high as 10 cm. Small variation (0.021 m/s) in speed of the fibre fuse owing to the laser power fluctuation has been observed. Since the Doppler broadening was less than 2 kHz in our system, the achieved resolution for speed measurement is as high as 1 × 10^−3^ m/s. As an example of its application, we studied instantaneous speeds in propagating fibre fuses and their power dependences using different optical fibres. To the best of our knowledge, it is the first report on the real-time measurement of the propagation speed of a fibre fuse. Our method is promising for both real time fibre fuse monitoring applications and future studies on its propagation and termination mechanism.

The OFDR uses the frequency-modulated continuous wave (FMCW) ranging technique, referring to the radar technology^21^. It was proposed and developed for optical network diagnostic application to overcome the spatial resolution limitation of OTDR. The key component of OFDR is a linearly swept laser with a constant frequency tuning speed *γ* and the spatial resolution of it is mainly determined by the frequency sweeping range^22^. The lightwave of the laser is divided into the reference beam and the probe beam by a fibre coupler. The backscattered/reflected probe light interferes with the reference light and any time delay (*τ*) caused by the optical path difference between them generates a beat frequency (∆*f* ) with a relationship of ∆*f* = *γτ*. The OFDR trace is produced by using Fourier transform to convert optical frequency domain response to the desired spatial and intensity response. The equivalent distance *Z* of the reflection point can be expressed as


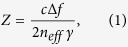


where, *c* is the speed of light in vacuum, ∆*f* is the beat frequency acquired from the data acquisition device, *n*_*eff*_ is the effective group index of the fibre.

During the propagation of a fibre fuse, the reflection point from the boundary of the hot plasma moves towards both the transmitter (linearly swept laser) and the receiver (photo detector) in our system, which introduces a change in the frequency as a result of the Doppler shift as shown in Fig. 1. The relationship between the Doppler shift *f*_*Doppler*_ and the fibre fuse propagation speed *v* is expressed as:


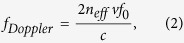


where, *f*_*0*_ is the frequency of the probe light. *f*_*Doppler*_ can be obtained from the difference between the beat frequency ∆*f*_2_ and ∆*f*_1_. As a result, the propagation speed of a fibre fuse is expressed as:


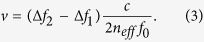


In experiment, ∆*f*_2_ can be acquired from the data acquisition device and ∆*f*_1_ is only related to the equivalent distance of the reflection point. By assuming that *Z* of the first sampling point after fuse initiation is equal to the initial length of the fibre, *v* at this point can be obtained from equation (2) and equation (3). Moreover, because the fibre fuse propagation speed change within a single sampling interval *T* is negligible, *Z′* of the next sampling point can be expressed as *Z′* = *Z* − *vT*. By repeating these procedures, *Z* and *v* at any sampling point can be calculated during fuse propagation. The resolution for speed measurement is determined by the spatial resolution of OFDR and the Doppler broadening which influences ∆*f*_2_.

## Results and Discussion

### Fibre fuse detection

**Figure 2:**
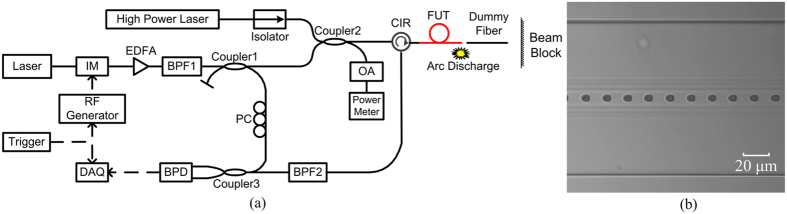
(**a**) Experimental setup. IM: intensity modulator; EDFA: Erbium-doped optical fibre amplifier; BPF: bandpass filter; OA: optical attenuator; CIR: circulator; PC: polarization controller; BPD: balanced photo detector; DAQ: data acquisition; Coupler 1 and Coupler 3: 3 dB coupler; Coupler 2: 10 dB coupler; FUT: fibre under test; (**b**) Micrograph of the FUT after fuse propagation.

**Figure 3:**
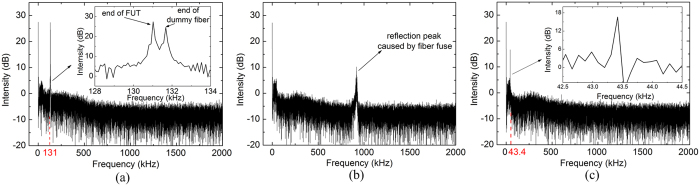
OFDR traces of the FUT: (**a**) before the fibre fuse initiation; (**b**) during the fibre fuse propagation; (**c**) after the fibre fuse termination. The beat frequency of Fresnel peaks before and after the fibre fuse initiation are depicted with dotted lines in (**a**,**c**), respectively.

We experimentally demonstrated real-time locating and speed measurement of fibre fuse using the proposed method. The experimental setup is shown in Fig. 2(a), and its detailed information is given in Methods section. We used a pure silica-core SMF (Corning SMF-28^®^ ULL optical fibre) as the fibre under test (FUT) for fibre fuse detection and the optical power coupled into it was about 3.3 W. Figure 3(a–c) show the OFDR traces of the FUT before the initiation, during the propagation and after the termination of the fibre fuse, respectively. The OFDR traces were measured without averaging process in order to achieve the quickest fibre fuse detection. Before the fibre fuse was initiated, besides the Fresnel reflection peak representing the end of the FUT, an additional peak were observed as shown in Fig. 3(a). This was due to the existence of a piece of dummy fibre employed for safety purposes. The fibre fuse was initiated at the end of the FUT, which corresponds to a beat frequency about 131 kHz and its equivalent distance calculated from equation (1) was 67.2 m. After the fibre fuse was initiated, the beat frequency of reflection peak instantaneously jumped from 131 kHz to a higher frequency (about 900 kHz) and the intensity of the reflection peak became lower than its original level. This feature is employed to detect the start of a fibre fuse.

The response time for the fibre fuse detection, which refers to the required time for the change from the state of Fig. 3(a) to Fig. 3(b), is mainly determined by the time for the data acquisition (DAQ), which did not exceed 200 ms in our experiment. Since the fibre fuse propagation speed was lower than 0.5 m/s with a launched power of about 3 W, the fibre fuse can be detected and terminated within a propagation distance of 10 cm. The response time can be further reduced by improving the DAQ performance. Since the response time is theoretically independent to the fibre length, our method for fibre fuse detection can be applied to fibre links longer than the laser coherence length by employing phase noise compensated OFDR^23^.

### Location and speed measurement

The position of the last void created by the fibre fuse in the damaged fibre was accurately located after the fibre fuse was terminated. When we turned off the high power laser and terminated the fibre fuse, the frequency shift due to the Doppler Effect also disappeared immediately. The original reflection peak at 131 kHz representing the end point of the FUT moved to the frequency representing the place of the last void as shown in Fig. 3(c). We observed a reflection peak at 43.4 kHz corresponding to the equivalent distance of 22.3 m, which indicated that 44.9-m-long fibre had been destroyed during the fibre fuse propagation. Our method achieved a locating accuracy as high as about 10 cm owing to the high spatial resolution of OFDR. The estimated average propagation speed of the fibre fuse was 0.42 m/s according to the propagation time of 106 seconds recorded by a camera.

**Figure 4:**
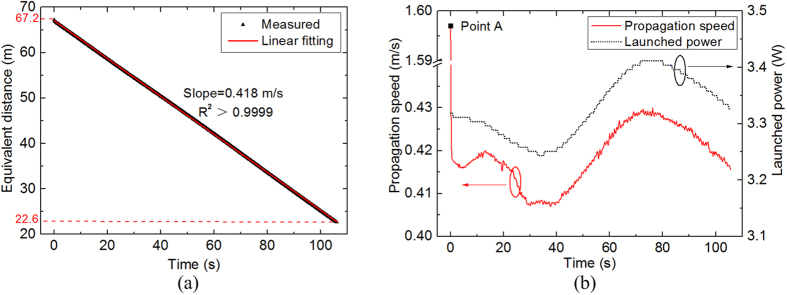
Real-time locating and speed measurement of fibre fuse in a pure silica-core SMF: (**a**) equivalent distance of the last void as a function of time; (**b**) fuse propagation speed and launched power as functions of time.

We real-time measured the location of the plasma of a propagating fibre fuse with a constant launched power and the results are shown in Fig. 4(a). We acquired 530 points as the fibre fuse propagating towards the light source and the location of the fibre fuse progressed linearly from 67.2 to 22.6 m. The average propagation speed of the fibre fuse estimated from the fitting line in Fig. 4(a) is 0.418 m/s with a coefficient of determination R^2^ higher than 0.9999. The calculated position of the final leading void was 22.6 m, which agrees well with the result estimated from the OFDR trace acquired after the fibre fuse termination.

The propagation speed of the fibre fuse and launched power in the FUT as functions of time are shown in Fig. 4(b). We observed a subtle fluctuation (5.1%) in fibre fuse propagation speed from 0.408 to 0.429 m/s. Its trend agrees well with that of power fluctuation (5.2%) in the FUT owing to the laser source instability. Since the spatial resolution and the Doppler broadening are less than 10 cm and 2 kHz, respectively, the derived resolution for speed measurement using equation (3) is 1 × 10^−3^ m/s. In addition, the speed of the first measuring point A is about 3 times faster than the following points. It is because that point A presents fibre fuse propagation speed in the 30-cm-long dispersion compensating fibre (DCF) at the end of the FUT, which has a smaller MFD than that of the FUT. To the best of our knowledge, it is the first report on the real-time measurement of propagation speed of a fibre fuse.

**Figure 5:**
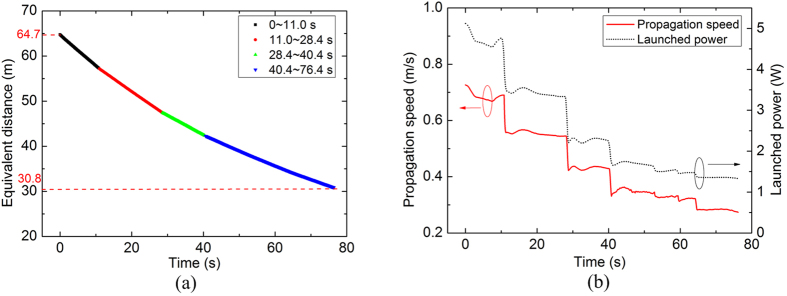
Real-time locating and speed measurement of fibre fuse in a Ge-doped SMF: (**a**) equivalent distance of the last void as a function of time; (**b**) fuse propagation speed and launched power as functions of time.

In order to investigate the relationship between the propagation speed of the fibre fuse and the launched power in a wide power range, we carried out fuse monitoring experiment using a Ge-doped core SMF (YOFC FullBand^®^ Plus Fibre) by gradually decreasing the launched power during the fibre fuse propagation. The launched power was gradually decreased from 4.6 W to about 3.3 W, 2.2 W, and 1.5 W at 11.0, 28.4, and 40.4 seconds, respectively. The equivalent distance during each time interval are plotted in black, red, green and blue colour, respectively, in Fig. 5(a). It can be observed that the slope representing the propagation speed is gradually decreasing. The fibre fuse propagation speed and launched power as functions of time are shown in Fig. 5(b). When the launched power was decreased 17% from 4.2 to 3.5 W, the fibre fuse propagation speed decreased 20% accordingly from 0.690 to 0.551 m/s. The curve representing the propagation speed shows excellent similarity with that representing the launched power.

**Figure 6:**
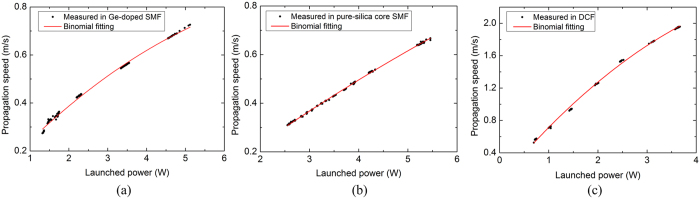
Fibre fuse propagation speed as functions of the launched power in different fibres: (**a**) Ge-doped SMF (**b**) pure-silica core SMF (**c**) DCF.

We studied the fibre fuse propagation characteristics of different kinds of optical fibres using the proposed method. Figure 6(a–c) show the relationship between the propagation speed and launched power in Ge-doped SMF, pure silica-core SMF and DCF, respectively. In experiments, the launched power was gradually decreased until the fibre fuse terminated. It can be observed that at a same launched power, 3.5 W for example, the propagation speed of Fibre A, B, and C are 0.562, 0.430, and 1.902 m/s, and their threshold power for fuse propagation are 1.3, 2.5, and 0.6 W, respectively. Although both Fibre A and Fibre B complies with ITU-T recommendation G.652, Fibre B has a higher threshold power and a lower fuse propagation speed at a same launched power. There are two possible reasons for this phenomenon. One reason is that the Fibre B may have a slightly larger MFD; the other reason is that the fibre with a pure silica-core is more immune to the fibre fuse according to the Ge-related defects proposed by Hand^2^. In addition, the fibre fuse propagation speed as a function of the launched power exhibits nonlinearity which is more obvious in Fibre C with a smaller MFD. This effect has been studied using a one-dimensional heat conduction model^24^. However, the model does not fit well with our experiment results and a more complicated model taking the mode field distribution into consideration is necessary for the further study.

Moreover, it should be noticed that in order to employ our method for fibre fuse detection, it is important to obtain the position of the initial point of a fibre fuse in order to estimate both its real-time location and the speed. This can be realized by detecting the changes in local loss of the fibre. Before the start of a fibre fuse, the local temperature of the affected point increases to an order of 10^3^ K, and the absorption coefficient of the point largely increases with the temperature^2^,^25^. This causes a dramatic increase in the local fibre loss which can be observed by OFDR with improved signal-noise ratio (SNR). Our method will be a convenient tool which provides more possibilities for the further studies on fibre fuse phenomenon.

## Conclusion

We propose and experimentally demonstrate real-time location and speed measurement of the fibre fuse using optical frequency-domain reflectometry. We successfully detected the start of the fibre fuse within a propagation distance of about 10 cm in a 67 m-long SSMFs. After the fibre fuse was terminated, the last void of the damaged fibre was located with resolution as high as 10 cm. The response time for fuse detection (within 200 ms in our demonstration) can be further reduced by improving the data acquisition and processing techniques. Since the response time is theoretically independent to the fibre length, our method is suitable for fibre links with a long length.

We successfully measured instantaneous speed of the propagating fibre fuse and observed subtle fluctuation in speed owing to the laser power instability. The resolution for speed measurement achieved in our demonstration is 1 × 10^−3^ m/s. We studied the fibre fuse propagation speed dependence on the launched power in different fibres and confirmed that optical fibres with a pure silica-core and a larger mode field diameter are more immune to the damage of fibre fuse. Our method is promising for both real time fibre fuse monitoring applications and future studies on its propagation and termination mechanism.

## Methods

The proposed fibre fuse monitoring system using OFDR is shown in Fig. 2(a). A high power fibre laser (Keopsys, ML20-CW-R-TKS-1550) at a wavelength of 1549.7 nm was used as the light source to initiate fibre fuse and about 90% of the power from it was coupled into the FUT using a 10 dB fibre coupler (Coupler 2). The optical power in the FUT was monitored by measuring the power of the other port of Coupler 2 using a power meter with a 20 dB optical attenuator. Another laser (NKT, E15) operating at 1550.3 nm with a narrow linewidth of 1 kHz was used as the light source for OFDR, and the light from it was modulated by an intensity modulator (Optilab IM-1550-20-A). The RF generator (Agilent E8257D) provided frequency sweep signals ranging from 17 GHz to 19 GHz and the sweep speed was 200 GHz/s. The modulated light was amplified by an Erbium-doped optical fibre amplifier (EDFA) and filtered by a tuneable bandpass filter (BPF1) to create signals with the desired lower side band. After that, the modulated light was divided into two beams using a 3 dB fibre coupler (Coupler 1). One beam was used as the local reference light and the other was used as the probe light. The probe path was connected to the 10% port of Coupler 2. We used a tuneable bandpass filter (BPF2) to eliminate both the backreflected and backscattered light from the high power laser. The Rayleigh scattered and the reflected light from the FUT was coherently detected with the local reference. The beat frequency signals were collected at a sampling rate of 8 MS/s using a DAQ (NI PXI-5922) and the sampling interval was 200 ms.

In order to initiate the fibre fuse easily, we spliced a piece of dispersion compensating fibre (YOFC DM1010-A) with a MFD of 5 μm at the end of the FUT and used an arc discharge to initiate the fibre fuse. The fibres used in our experiments are YOFC FullBand^®^ Plus Fibre, Corning SMF-28^®^ ULL and YOFC DM1010-A.

## Additional Information

**How to cite this article**: Jiang, S. *et al*. Real-time locating and speed measurement of fibre fuse using optical frequency-domain reflectometry. *Sci. Rep*. **6**, 25585; doi: 10.1038/srep25585 (2016).

## Figures and Tables

**Figure 1:**
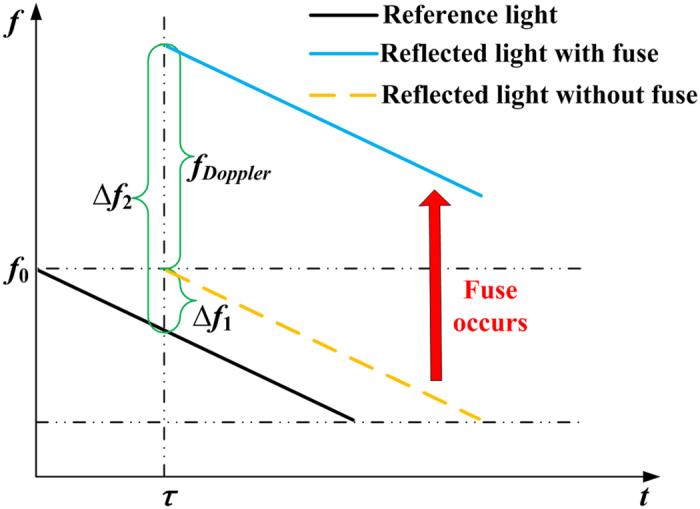
A schematic of frequency shift introduced by the fibre fuse.

## References

[b1] Kashyap R. & Blow K. J. Observation of catastrophic self-propelled self-focusing in optical fibres. Electron. Lett. 24, 47–49 (1988).

[b2] Hand D. P. & Russel P. S. J. Solitary thermal shock-waves and optical damage in optical fibres: the fiber fuse. Opt. Lett. 13, 767–769 (1988). 19746030 10.1364/ol.13.000767

[b3] Kashyap R. The fiber fuse - from a curious effect to a critical issue: A 25th year retrospective. Opt. Express 21, 6422–6441 (2013). 23482212 10.1364/OE.21.006422

[b4] Dianov E. M., Bufetov I. A. & Frolov A. A. Catastrophic damage in specialty optical fibers under CW medium-power laser radiation. J. Opt. (India) 33, 171–180 (2004).

[b5] Todoroki S. Origin of periodic void formation during fiber fuse. Opt. Express 13, 6381–6389 (2005). 19498651 10.1364/opex.13.006381

[b6] Rocha A. M., Domingues F., Facao M. & Andre P. S. Threshold power of fiber fuse effect for different types of optical fiber. In *13th International Conference on Transparent Optical Networks* (Stockholm, Sweden, 2011), paper Tu. P. 13.

[b7] Bromage J. Raman amplification for fiber communications systems. J. Lightwave Technol. 22, 79–93 (2004).

[b8] Kahn J. M. & Ho K. P. A bottleneck for optical fibres. Nat. 411, 1007–1010 (2001). 10.1038/35082671 11429585

[b9] Richardson D. J., Fini J. M. & Nelson L. E. Space division multiplexing in optical fibres. Nat. Photonics 7, 354–362 (2013).

[b10] Puttnam B. J. . 2.15 Pb/s transmission using a 22 core homogeneous single-mode multi-core fiber and wideband optical comb. In *41st European Conference on Optical Communication* (Valencia, Spain, 2015), PDP. 3. 1.

[b11] Jackson S. D. Towards high-power mid-infrared emission from a fibre laser. Nat. Photonics 6, 423–431 (2012).

[b12] Cesar J., Jens L. & Andreas T. High power fiber lasers. Nat. Photonics 7, 861–867 (2013).

[b13] Zhang H., Zhou P., Wang X., Xiao H. & Xu X. Fiber fuse effect in high-power double-clad fiber laser. In *11th Conference on Lasers and Electro-Optics Pacific Rim* (Kyoto, Japan, 2013), paper WPD. 4.

[b14] Dianov E. M., Bufetov I. A. & Frolov A. A. Destruction of silica fiber cladding by the fuse effect. Opt. Lett. 29, 1852–1854 (2004). 15357337 10.1364/ol.29.001852

[b15] Rocha A. M., Fernandes G. & Domingues F. Halting the fuse discharge propagation using optical fiber microwires. Opt. Express 20, 21083–21088 (2012). 23037231 10.1364/OE.20.021083

[b16] Hanzawa N. . Suppression of fiber fuse propagation in hole assisted fiber and photonic crystal fiber. J. Lightwave Technol. 28, 2115–2120 (2010).

[b17] Abedin K. S., Nakazawa M. & Miyazaki T. Backreflected radiation due to a propagating fiber fuse. Opt. Express 17, 6525–6531 (2009). 19365477 10.1364/oe.17.006525

[b18] Abedin K. S. & Nakazawa M. Real time monitoring of a fiber fuse using an optical time-domain reflectometer. Opt. Express 18, 21315–21321 (2010). 20941027 10.1364/OE.18.021315

[b19] Dianov E. M. . High-speed photography, spectra, and temperature of optical discharge in silica-based fibers. IEEE Phot. Tech. Lett. 18, 752–754 (2006).

[b20] Rocha A. M., Antunes P. F. C., Domingues F., Facao M. & Andre P. Detection of fiber fuse effect using FBG sensors. IEEE Sensors J. 11, 1390–1394 (2011).

[b21] Fang Z., Chin K. K., Qu R. & Cai H. Fundamentals of optical fiber sensor (ed. Chang K.) 298–299 (Wiley, New Jersey, 2012).

[b22] Ghafoori-Shiraz H. & Okoshi T. Optical frequency-domain reflectometery. Opt. Quantum Electron. 18, 265–272 (1986).

[b23] Fan X., Koshikiya Y. & Ito F. Phase-noise-compensated optical frequency-domain reflectometry. IEEE J. Quantum Electron. 45, 594–602 (2009).

[b24] Ankiewicz A., Chen W., Russell P. S. J., Taki M. & Akhmediev N. Velocity of heat dissipative solitons in optical fibers. Opt. Lett. 33, 2176–2178 (2008). 18830343 10.1364/ol.33.002176

[b25] Dianov E. M. . Catastrophic destruction of fluoride and chalcogenide optical fibers. Electron. Lett. 38, 783–784 (2002).

